# A systematic literature review on persuasive technology at the workplace

**DOI:** 10.1016/j.patter.2022.100545

**Published:** 2022-08-12

**Authors:** Kilian Wenker

**Affiliations:** 1 Friedrich-Alexander-Universität (FAU), Erlangen-Nürnberg, Germany

**Keywords:** persuasive technology, human-computer interaction (HCI), behavioral change, digital workplace design, gamification

## Abstract

Employees face decisions every day—in the absence of supervision. The outcome of these decisions can be influenced by digital workplace design through the power of persuasive technology. This article provides a structured literature review based on recent research on persuasive technology in the workplace. It examines the design and use of persuasive systems from a variety of disciplinary perspectives and theories. The reviewed studies were categorized into the research streams of technology design, user-centered research, and gamification. The purpose of the studies is categorized using a modified definition of the persuasive systems design model. A number of experimental studies show that alignment of the employee’s behavior with the employer’s agenda can be achieved. A robust finding is the key role of interactivity in granting employees a subjective experience of rapid and meaningful feedback when using the interface.

## The bigger picture

Persuasive technology aims to change human behavior through the use of computer technology. This interdisciplinary field of research is becoming increasingly popular and is being applied in a growing number of areas, from health to the environment to education. The field of gamification, which uses game elements outside of games, is growing particularly fast. While persuasive technology tends to use cognitive approaches to persuade people to behave in a certain way, gamification tries to do the same in a more playful, affective way.

This review presents key concepts related to persuasive technology in the workplace. For instance, interactivity facilitates feedback for employees by providing quick and compelling messages and prompting the employee to respond to persuasive technology. Rapid data collection and analysis reinforce this feedback. Positive incentives motivate and engage employees, especially when feedback is meaningful and personalized.

## Teaser

Persuasive technology has the potential to change everyone’s lives. This study focuses on persuasive technology in the workplace. It highlights the overlap with gamification and filters out which mechanisms impact the success of persuasive technologies.

## Introduction

Persuasive technology (PT) refers to interactive information technology designed for changing users’ attitudes or behavior in the field of human–computer interaction (HCI).^1^ With its manipulative and often invisible power to influence employees, it might be game changing for digital workplace design.

Take Microsoft as an example, where Ross Smith leads a team of testers who put unified communications products through the paces to find defects. When it was hard to find enough employees around the world willing to review Windows dialogue boxes in their spare time, Ross Smith invented a language quality game and made employees compete against each other to win the most points—making employees go above and beyond their work responsibilities.^2^^,^^3^

Incentives work both ways, with punishment instead of a positive reward. Take, for example, a medium-sized call center in Germany with several branches that take inbound telephone calls for customer service and sales. If a customer wants to cancel his subscription, the employees shall dissuade the customer from unsubscribing in a so-called retention attempt. A discount may be offered for this purpose as a last resort. However, many employees tend to give this discount liberally since it increases their customer retention rate and simplifies the phone call. This in turn hurts the company’s profit margin. The work flow software has been rewritten to introduce a random chance of blocking the discount. This makes the employee wait until the very end before offering a discount. What happens here is ultimately an automated, somewhat forced change in behavior, because an employee promising the discount without actually being able to grant it at the end of the call has a much more difficult conversation afterward with the customer, who will certainly cancel after a disappointed expectation. Here, PT comes with discouragement and repetition; the discouragement is in the form of harmful consequences as opposed to a reward and the repetition is an effective instrument of persuasion.

PT creates opportunities for various organizational needs. It may persuade customers if you consider one-click checkouts or purchase recommendations on websites like amazon.com. Or it may help managers to persuade employees like in the two workplace examples above. My research goal is to provide an overview and classification of recent research on PT at the workplace. To reach this goal, I perform a literature review following the principles laid down by Webster and Watson.^4^

The rest of this article is organized as follows: a section on theoretical background establishes the theoretical background underlying my systematic review; a section on research methods explains the literature review process; the results section summarizes the main findings thereof; and the discussion section discusses the implication of those findings. Finally, the conclusion provides a summary of these findings.

## Theoretical background

Workplace design has significantly changed since human work has been increasingly disrupted or even determined by information and communication technologies. An HCI environment necessitates a deep understanding of various disciplines, since designing digital technology for a group of employees involves psychology (how humans behave as individuals), sociology (how humans behave in a group), computer science (how to develop software), organizational studies (how organizational structures, processes, and practices work), and information systems (how to develop, use, and apply information technology in the business).^5^

The term persuasion refers to an attempt to shape, reinforce, or change behaviors, feelings, or thoughts about an issue, object, or action.^1^ PT is broadly defined as technology that aims to change user behaviors or underlying attitudes.^1^^,^^6^, ^7^, ^8^ This concept has recently been challenged, because PT raises questions around the borderlines between encouragement, persuasion, and, in particular, coercion.^9^ Advocates of the classical definition (which in general excludes coercion) suggested the term behavior change support systems.^10^ Other researchers started explicitly avoiding the term PT and suggested using behavior change technologies instead, explicitly including coercion.^11^ This article includes coercion to incorporate a setting such as the call center mentioned in the introduction, but will stick with the term PT.

As might be expected from an interdisciplinary field like HCI, the research has split in two directions: the technological design, which focusses on the technological aspects; and user-centered research, which encompasses the human aspects.^12^ Most research focusses on the latter. However, these two streams paint only a partial picture.

Gamification is generally understood as the use of game design elements and principles to make everyday tasks more engaging—but in non-gaming contexts.^13^^,^^14^ The mechanisms that motivate and engage players can also be used in other areas, such as motivating and engaging employees in the workplace. Thus, gamification is part of the definition of PT that this article uses, if technology is involved and the aim is to change user behaviors or underlying attitudes. A more nuanced view highlights that if employees cannot turn off the game, then it is no longer a game.^15^

However, while PT often lacks the actionable implementation and instead lists persuasive affordances or general design principles, gamification offers a plethora of effective implementation mechanisms and does not focus on the underlying theory. Take the persuasive affordance of “self-surveillance”^1^ or “self-monitoring.”^8^^,^^16^ In a game, the player’s score and all performance parameters and achievements are tracked and displayed using a scoreboard. Gamification replicates this scoreboard mechanism in the world of work to induce behavior change. While PT focuses on self-monitoring (also called feedback) as a design principle, gamification focusses on the scoreboard. Or, as another example, take the persuasive effects of “reminders”^8^^,^^16^ or “self-efficacy.”^1^ In a game, when a player accesses a frequently visited, central menu in a game, this menu always contains a reminder pointing to the main task at that point, so that the player does not deviate from the main tasks too far, for example, by pursuing bonus activities. Gamification in the workplace, in this sense, would be if a dashboard always displayed the employee’s current quarterly goal. As employees access the dashboard to see, for example, the number of tickets they have completed, they are always reminded of their main goal and encouraged to change their behavior to work more concretely toward their main goal, which might be decreasing the average time spent working through tickets.

Gamification lacks a body of literature explaining its inner workings, because of its origin and source of inspiration, games. Games have evolved over centuries, with the most entertaining ones prevailing while the less entertaining ones have perished—not through theory, but through practice. This process of survival of the fittest has led to games being very sophisticated and the adaption of gamification mechanics in work environments and other domains like health and education.

**Figure 1:**
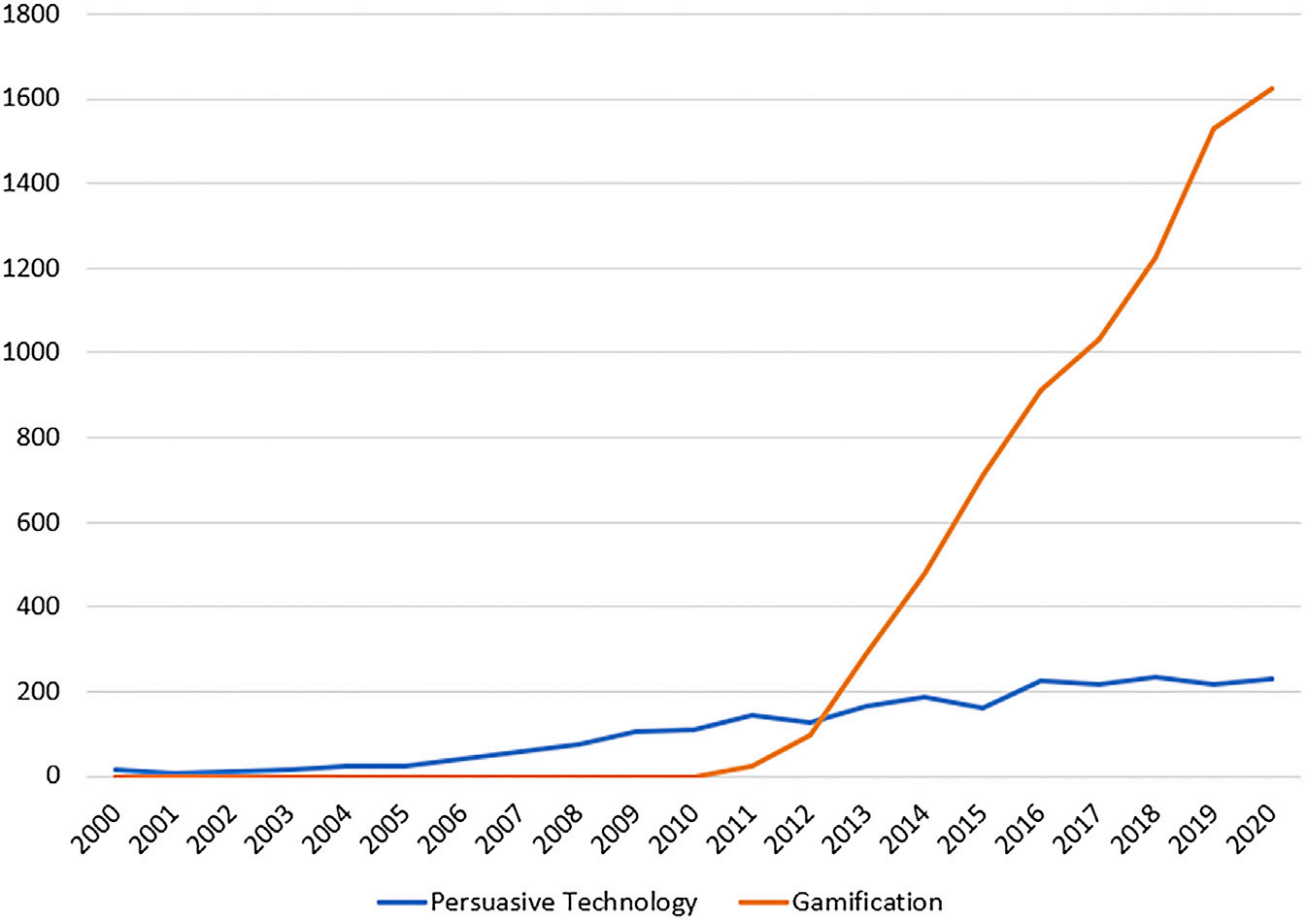
Number of search hits, by year, for the queries (TITLE-ABS-KEY(gamification) AND PUBYEAR >1999) and (TITLE-ABS-KEY(Persuasive technology) AND PUBYEAR >1999) in the Scopus database Adapted from Hamari et al.^7^

Figure 1 depicts how the amount of writing on PT has been increasing steadily. The graph shows that, approximately 10 years ago, the number of studies on PT first began to stagnate. What stands out in this chart is a sharp increase in the number of studies related to gamification at around the same time.

Previous research on PT has emphasized the importance of designing persuasion into technologies. The persuasive systems design (PSD) model represents the main conceptual framework on PT.^8^, ^9^, ^10^^,^^16^ It was specifically developed for HCI and distinguishes three categories, namely, understanding key issues behind persuasive systems, analyzing the persuasion context, and the design of system qualities. These three categories collect various elements, which are interrelated and overlap, and are formulated in very general terms.

Leaving the technological aspect aside, behavioral theory inevitably comes to the fore. The theory of planned behavior (TPB) is a psychological theory that states that three interrelated core factors, namely attitude, subjective norms, and perceived behavioral control, determine human intentions. Learning theory seeks to describe how students receive, process, and retain knowledge, influenced by cognitive, emotional, and environmental influences, as well as prior experience. As a consequence, learning is considered to be an aspect of conditioning, and a system of rewards and goals would be helpful.

Further prominent theories related to behavior change include the theory of reasoned action, the technology acceptance model, the self-efficacy theory, social cognitive theory (SCT), the elaboration likelihood model, the cognitive dissonance theory, the goal-setting theory and computer self-efficacy^11^^,^^17^, ^18^, ^19^ (see^10^ for an overview of these and further theories).

The theory of reasoned action assumes that a person’s actual behavior is determined by his or her behavioral intention. The latter, in turn, is derived from personal attitudes toward the intended behavior and from subjectively perceived norms. The technology acceptance model is more specific to the use of technology and sees perceived usefulness and perceived ease of use as the two main influences on behavioral intention (to use a new technology). Self-efficacy theory is often used as a subset of larger models. It proposes that all actions and their consequences form a feedback loop in which a person’s perceived abilities for a particular task are iteratively adjusted to previous experiences, thus influencing behavior. SCT is an example of a theory that extends self-efficacy. SCT states that an individual’s acquisition of knowledge may be directly related to the observation of others in the context of social interactions, and that this information may (or may not) lead to changes in behavior. The elaboration likelihood model bisects the way a persuasive communication is processed and infers whether enduring views are formed. The theory of cognitive dissonance examines the perception of contradictory information and various ways of overcoming these contradictions. The core idea of goal-setting theory is that goals motivate people and that setting goals means that a person focuses mentally, emotionally, and through his or her behavior on achieving the goal. Computer self-efficacy theory applies self-efficacy to computer use and emphasizes how—in terms of broader tasks—individuals’ expectations of their abilities influence their behavior in accomplishing those tasks.

Roughly summarized, all of these theories strive to explain how self-efficacy expectations and outcome expectations influence behavior. Once one has identified the causes of behavioral decisions, one can also try to change employee behavior by influencing the causes. Most theories address universal causality. A counterexample is computer self-efficacy theory, which focuses specifically on computer use.

## Research method

The review process began with the selection of the databases to be used for the literature searches. I chose the databases Scopus, Business Source Complete, and Web of Science. For Scopus, I conducted a keyword search formulating the following query: (TITLE-ABS-KEY(persuasive AND systems AND behavior) OR TITLE-ABS-KEY(behavior AND change AND support) OR TITLE-ABS-KEY(digital AND work AND design AND behavior)) AND PUBYEAR >1999 AND (LIMIT-TO (SUBJAREA, “COMP”) OR LIMIT-TO (SUBJAREA, “ENGI”) OR (SUBJAREA, “SOCI”) OR LIMIT-TO (SUBJAREA, “BUSI”) OR LIMIT-TO (SUBJAREA, “PSYC”) OR LIMIT-TO (SUBJAREA, “MULT”)) AND (LIMIT-TO (LANGUAGE, “English”) OR LIMIT-TO (LANGUAGE, “German”)). I performed the search in May 2021, which resulted in more than 82,000 hits. Next, I customized those results with a post-query filter by filtering the articles that have been cited at least 150 times. That second step decreased the number hits drastically to 171.

The second database I queried is Business Source Complete, using the following search terms: “work design” AND “persuasive.” Those were limited to “Peer Reviewed Journals” and narrowed by subject “persuasive technology.” The search mode I applied was “SmartText Searching.” Additionally, I expanded the query using internal synonyms to include “equivalent subjects.” From this, Business Source Complete returned 19 results.

I used a third database, Web of Science, which focusses on technical aspects. It provided a coverage of 240 articles in total, eight of which have been cited at least 150 times.

**Figure 2:**
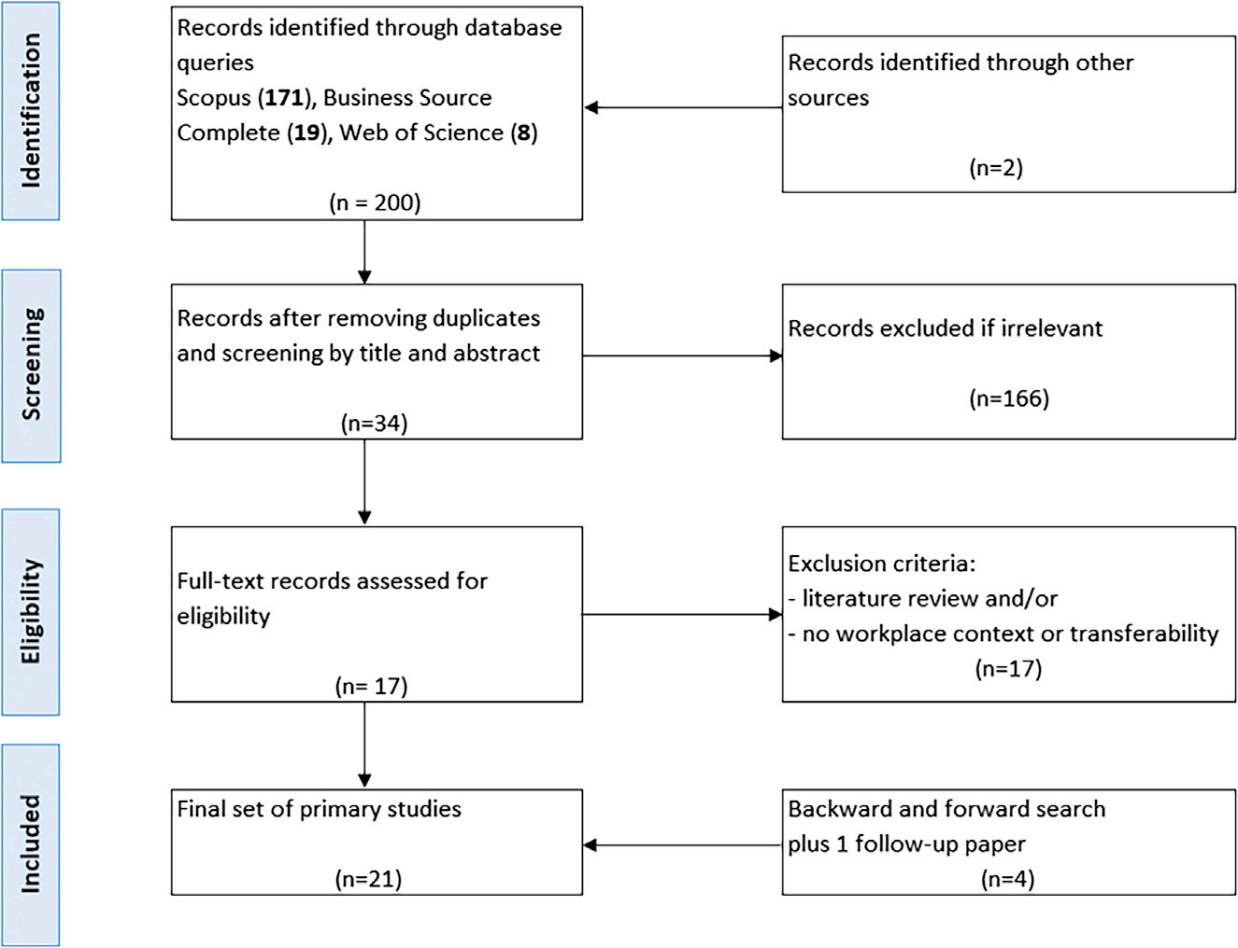
Flow diagram for study selection Adapted from Johnson et al.^20^

Next, I removed duplicates and filtered the titles and abstracts for relevant articles, which resulted in 34 hits. Full-text analysis served to assess the content of the remaining articles. A quality assessment of the 34 primary studies eligible for review was undertaken in parallel with the creation of a concept matrix. Figure 2 illustrates how 200 articles fit the initial inquiry, and how that number decreased to the final sample of 21 articles.

One of the articles found through backward citation searching^21^ describes the development of a social media application that promotes energy awareness while motivating employees to engage in energy-saving behaviors. In the follow-up publication,^22^ usability tests were conducted with 128 subjects as proxies for typical office building users. Since the results of that study were published across two separate research articles, I considered them to represent one primary study.

To classify the content of research, I allocate the three historic research streams of technological design, user-centered research, and gamification to the general direction of the research reviewed. Another way to break down the content is to draw a distinction between the three phases of the PSD framework. But since the three terms are more like catalog headings, I decided to redefine them to reflect the purpose of the study as follows. For understanding key issues, these articles aim to understand the fundamentals behind persuasive systems, whether they are technical or user centered. For analyzing the persuasion context, articles in this category focus on a concrete, working, or applied PSD. The focus may be analyzing the PSD in its entirety or investigating parts of it like the persuader, the persuadees, the intent, the strategies, technology-dependent features, and other aspects. For the design of system qualities, research in the second category provides information on content and functionality; there is a growing body of literature that recognizes the further development of existing possibilities. This research focusses on improving or evaluating an existing PSD.

A second concept matrix assesses the scientific approach of the reviewed articles, distinguishing studies focusing conceptual work, quantitative research and qualitative research. I classify the research design into laboratory experiments, crowdsourcing, in-field experiments and automated evaluation procedures (based on^23^).

As the name implies, post hoc explanations represent an attempt to make sense of the collected data after the fact. Statistical analysis has been instrumental in our understanding of the interdependence of actions, cognitions, and emotions. However, it is hard to tell what exactly is cause and effect. Observational or descriptive explanations strictly adhere to the data and refrain from interpreting what might be cause or effect. Although presume and assume both mean to assume something to be true, an assumption suggests there is little or no evidence supporting your guess, whereas a presumption implies greater confidence or evidence-based reasoning.^24^ In this article, I use the term explanatory assumptions if authors suppose a relation or conclusion without providing evidence.

## Results

The studies represent a range of different domains, including theoretical frameworks, empirical studies, and four case studies. The most widely represented domain was sustainability in the workplace (including energy savings). One study is notable for the unique persuasive approach of negative emotion, i.e., perceived threat from malicious information technology (IT).^19^ Most studies focus on the individual and describe the work setting, but include little context on the technology itself. Table 1 displays a concept matrix with all identified concepts for the content of the researched studies—the headings for those concepts being research stream and purpose of the study as presented in the Theoretical background section.

**Table 1:** Concept matrix for the content of the reviewed literature

Article	Reference	Research stream			Purpose of study		
		Technology design	User-centered research	Gamification	Understanding key issues	Analyzing the persuasion context	Design of system qualities
Johnston and Warkentin. (2010)	^19^	•	•		•		
Hekler et al. (2013)	^11^				•		
Kumar (2013)	^25^	•	•	•	•	•	
Makanawala et al. (2013)	^26^			•		•	
Oinas-Kukkonen (2013)	^10^	•	•		•	•	
Whitson (2013)	^15^	•	•	•	•	•	
Fritz et al. (2014)	^27^		•			•	
Lehrer et al. (2014)	^22^	•	•	•	•	•	
Lockton et al. (2014)	^28^		•	•		•	
Hamari and Koivisto (2015)	^18^		•	•	•		
Robson et al. (2015)	^14^		•	•	•	•	
Filippou et al. (2016)	^6^		•			•	
Chesney et al. (2017)	^29^		•		•		
Hamari (2017)	^30^		•	•	•	•	•
Liu et al. (2017)	^13^			•	•	•	
Stieglitz et al. (2017)	^31^		•				
Mogles et al. (2018)	^32^		•		•	•	
Khashe et al. (2019)	^33^		•			•	
Böckle et al. (2020)	^12^		•	•	•		•
Chiu et al. (2020)	^17^	•	•				•
Khataei et al. (2021)	^34^	•	•		•		

The studies represent a range of scientific approaches, including theoretical work, quantitative research, qualitative research, and a mixture thereof. Some theoretical or conceptual articles did not include any empirical data. The most popular discipline was quantitative research nonetheless. A common feature across many articles was the limited amount and quality of empirical evidence, often acknowledged by the authors themselves in the disclosed limitations of their studies. Table 2 displays a concept matrix with all identified concepts. These concepts are summarized under the headings scientific approach, research design, and post hoc explanations (of results), as presented in the Research method section.

**Table 2:** Concept matrix for the scientific methodology of the reviewed literature

Articles	Reference	Scientific approach			Research design				Post-hoc explanation			
		Conceptual work	Quantitative research	Qualitative research	Laboratory experiments	Crowdsourcing	In-field experiments	Automated eval. procedures	Statistical analysis	Observational and/or descriptive	Explanatory presumptions	Explanatory assumptions
Johnston and Wa. (2010)	^19^	•	•				•		•		•	
Hekler et al. (2013)	^11^	•										
Kumar (2013)	^25^	•										
Makanawala et al. (2013)	^26^			•								
Oinas-Kukkonen (2013)	^10^	•										
Whitson (2013)	^15^			•			•					
Fritz et al. (2014)	^27^			•			•			•		
Lehrer et al. (2014)	^22^		•	•	•				•	•	•	
Lockton et al. (2014)	^28^		•	•			•		•	•	•	
Hamari and Koivisto (2015)	^18^	•	•				•		•		•	
Robson et al. (2015)	^14^	•		•								
Filippou et al. (2016)	^6^		•	•							•	•
Chesney et al. (2017)	^29^		•		•				•	•		
Hamari (2017)	^30^		•				•		•	•	•	
Liu et al. (2017)	^13^	•										
Stieglitz et al. (2017)	^31^			•				•		•	•	
Mogles et al. (2018)	^32^	•	•		•		•	•	•		•	
Khashe et al. (2019)	^33^		•		•	•			•		•	
Böckle et al. (2020)	^12^		•			•			•		•	
Chiu et al. (2020)	^17^		•				•	•	•	•		
Khataei et al. (2021)	^34^		•				•		•		•	

Research on PT is scant in workplace contexts and offers an excellent opportunity for future research. Therefore, I looked for PT in other settings, if those studies were fundamentally appropriate to understand persuasion in the workplace. For instance, in a study on nudging people into eco-friendly behavior, 30 students worked on computers without change in their usual computer use, but their computers’ electricity consumption was measured and eco-feedback used to persuade.^17^ There is no obvious reason why the basic causalities should work differently for employees at PCs than for students at PCs. Other researchers extend this view by using mainly undergraduates for a simulation and stating that those students are proxies for typical office building occupants.^22^ Researchers have been reflective and receptive to discussions on the wide use of students as experimental subjects and online panel data, gauging experiments against real-world behavior.^35^^,^^36^ In Figure 2, I refer to proxy samples as transferability (to the workplace area).

Four case studies deal with companies that use PT in different areas of their business. The UK government’s Department of Energy and Climate Change introduced PT and some gamification mechanics to make workplaces more sustainable and succeeded, e.g., in decreasing the size of office space by encouraging employees to move into a common office space at night.^28^ Another case study illustrates approaches to improve productivity, spirit, and engagement at work of customer service agents by introducing gamification into SAP Service OnDemand.^26^ An analysis of the results of PT and gamification in call centers explores why the latter work or do not work there.^15^ Finally, semi-structured interviews are used to gauge the impact of digital nudges at an automotive supplier.^31^

## Discussion

### Principles and strategies hide the lack of comprehensive models or theories

I refer to the latter as a comprehensive and well substantiated explanation of phenomena like PT. From my sample of 20 articles, I identified frameworks that mainly expose the strengths and weaknesses of existing theories and incorporate those into a more or less structured theoretical framework^13^^,^^16^^,^^32^ or compile a concise and quite comprehensive table of theories, listing only theories developed in social psychology, except for computer self-efficacy.^10^ Two information systems theories explicate the cause behind actions and behaviors.^13^ Media characteristics are concerned with users’ choice of a type of medium. In SCT, any observed behavior can change a person’s mindset (cognition), and human behavior is explained as a continuous interaction between cognitive, behavioral, and environmental influences.

The PSD framework is receiving attention as a key paradigm for PT research.^10^ However, it does not explain the causal relationships; it essentially lists 28 principles or strategies, leaving it unclear how these should be implemented in a particular user context.^32^ Nevertheless, I found the rough breakdown useful and, after modification, I was able to use it to narrow down content concepts.

Most studies do not try to elaborate a comprehensive PT model or theory, but rather list specific principles or strategies, often in a selective manner (see^1^ for an inventory of those principles or strategies). In summary, no consensual comprehensive theory has emerged so far.^18^ Authors typically assemble an eclectic mix of principles, taken from different theories, to design a persuasive system rather than design a comprehensive theory. And since, for instance, a system designed to assist in weight loss is not easily adaptable to convince people about energy conservation,^34^ one could argue a sound, generalized model would have to incorporate too many variables. In other words, the workings of behavior change are highly complex. Yet it is clear that the success of simple principles and strategies is what makes PT so appealing.

### Interactivity increases the persuasive effects of PT

The term interactivity is used to describe the communication process that takes place between humans and computer software, for instance, if a computer-based system expects a response from the user and provides feedback to that response. But to see user–system interactions as something that the user does is a rather limited view, given the level of interactivity involved in the current systems.^13^^,^^14^ The challenge of technology here is to provide a concrete cue for the behavior of interest, although a key question remains as to what form that cue should take, e.g., a motivational message, encouragement, reward, behavior prompts, reminders, or any other persuasive communication method.^32^

The literature affirms interactivity’s ability to create a highly engaging and cognitively engaging experience. According to the TPB, behavioral intentions are affected by attitude, subjective norms, and perceived behavioral control while the comprehensive action determination model points out that habits, intentions, and situations directly affect individual behavior.^17^

Persuasive strategies such as competition, self-monitoring and feedback, goal setting and suggestion, personalization, reward, and social comparison lead to different effects in distinctive situations and may drift, depending on the user’s mood and personality.^12^^,^^34^ An interactive social dialogue encourages users to comply more with requests when they otherwise might be more reluctant, a strategy that works differently across different types of people.^29^^,^^33^ Suggesting that interactivity plays a key motivational role in helping employees adjust their behavior is not new. What is new is how interactivity in the context of PT accelerates and amplifies this feedback by enabling rapid data collection and analysis, and a greater understanding and appreciation of the persuasive principles that shape human behavior. PT not only delivers quick and persuasive messages to the employee, but it creates an environment for the adoption of a particular action, i.e., the very act of using interactive PT privileges some options over others. There is a notable difference between, for instance, seeing and reading a generic advertisement on a website, a customized online advertisement based on browsing history and social media excerpts,^34^ and an interactive PT like a design pattern on a cookie consent request that nudges or even forces the user through a persuasive interface design.

Providing real-time feedback about employees’ actions by amassing large quantities of data and then simplifying these data into modes that are easily understandable, such as progress bars, graphs and charts may help employees or managers in gauging the situation. However, a potential source of bias is surveillance. Just because a PT is successful in gamified spaces does not mean that the same technology will be equally accepted and treated in other spaces. For gamification mechanics^14^^,^^25^^,^^28^^,^^30^ to be experienced as a fun way to work, all employees need to be willing participants.^15^

### Positive incentives are much more common than negative incentives

A key aspect for the application of PT in the workplace is to cause desired employee behavior. Several theoretical models specify the motivational and cognitive antecedents of behavior change, e.g., the theory of reasoned action,^8^^,^^10^^,^^17^^,^^19^ the TPB,^7^^,^^10^^,^^11^^,^^17^, ^18^, ^19^^,^^30^ or the theory of self-determination (SDT)^11^, ^12^, ^13^^,^^17^, ^18^, ^19^^,^^23^^,^^32^; see^37^ for an overview of how job motivation is affected by technologies and see^13^ for different theoretical perspectives on gamification research in the workplace.

Relying on SDT, and the distinction between intrinsic motivation (the drive to do something without external rewards and for its own sake) and extrinsic motivation (performing an activity to attain some separable outcome), the PSD framework proposes that rewarding target behaviors reinforces those behaviors and can increase the persuasiveness of a system, but it is the combination of rewards, collaboration, and competitive setting that is crucial.^8^^,^^13^^,^^14^^,^^20^^,^^30^ There is evidence of a strong relationship between positive external rewards and certain behavior outcomes.^20^^,^^22^^,^^25^^,^^26^^,^^30^ While PT without gamification elements aims to support decision-making through cognitive processes (i.e., the reward is extrinsic), gamification, in contrast, draws on affective processes, i.e., gamification is aimed at invoking employees’ or users’ intrinsic motivations through design reminiscent from games.^18^^,^^25^ If we look at gamification from the perspective of the traditional reward dichotomy, it would be difficult to categorize it as either intrinsic or extrinsic, since gamification provides both benefits: an external benefit such as task completion and an internal, hedonistic benefit such as fun. It is notable that making the consequences of a certain action visible to the employee, even in real-time PT, is not intrinsically meaningful information in itself. Employees would need a context like comparing the results with an objective or other employees’ results,^31^ or personalized incentive mechanisms.^12^

Negative incentives do exist, but are often hidden. Setting challenges and goals can be associated with different outcomes—positive if the employee succeeds or negative if the employee fails to reach the goal and that failure is measured and displayed. While many studies focus on positive rewards, the most notable exception explores user behavior in reply to negative rewards: malicious threats concerning IT security in the workplace inspire different outcomes for different users based on their perceptions of efficacity and threat, but combining threat severity with users self-efficacy and perceptions of response efficacity leads to more persuasive impact.^19^

Irrespective of the above, positive rewards may not have the desired effect on employee behavior. A misaligned reward system can lead to an employee gaming the system, at the expense of the employer.^6^^,^^13^^,^^14^^,^^27^ The same is true if system-based rewards exert a strong influence on people’s personal goals, to such an extent that the system goals seemed to supplant underlying goals, i.e., the artificial rewards gets more important than the real task.^6^^,^^27^

## Conclusion

The literature on PT strategies and principles continues to grow rapidly. Drawing on that literature, this survey presented key concepts related to PT in the workplace. Although there does not seem to be serious disagreement among researchers about the motivational and cognitive antecedents of behavior change, the findings highlight a gap in comprehensive theory building. Interactivity facilitates feedback for employees by providing quick and compelling messages and also by requiring the employee to respond to the PT. Since employees are receptive to rewards and competition in the workplace, the results of this review imply that positive incentives are mostly successful in motivating and engaging employees, especially if feedback is meaningful and personalized. The stimulus is transmitted in part through cognitive processes and, in the case of gamification mechanisms, through affective processes.

Since the reviewed studies vary in their methods and in the details of the research questions, and lack a comprehensible view, researchers should address this issue by integrating from a diverse set of technical, behavioral, organizational, and social disciplines (to name a few). Moreover, employee preferences should be addressed, since empirical studies in personalization imply that the effects of incentives may be considerably different than predicted by generic theories.^12^^,^^34^^,^^37^
